# Parents’ experiences of family and daily life after their child’s stay in the pediatric intensive care unit: a qualitative descriptive study

**DOI:** 10.1186/s12887-024-04883-z

**Published:** 2024-07-01

**Authors:** Sandra Stalder, Daniela Händler-Schuster, Franzisca Domeisen Benedetti

**Affiliations:** 1 School of Health Sciences, Institute of Nursing, ZHAW – Zurich University of Applied Sciences, Katharina-Sulzer-Platz 9, Winterthur, 8401 Switzerland; 2 Medical Informatics, and Technology UMIT, Department of Nursing Science and Gerontology, Institute of Nursing, Private University of Health Sciences, Hall in Tyrol, Austria; 3 Faculty of Health, School of Nursing, Midwifery and Health Practice, Te Herenga Waka - Victoria University of Wellington - Te Herenga Waka, Wellington, New Zealand

**Keywords:** Experience, Pediatric intensive care unit, Critically ill child, Follow-up, Parents, Family and daily life

## Abstract

**Introduction:**

The stay of a critically ill child in a pediatric intensive care unit (PICU) is a significant experience for the family. Thus far, little is known regarding the impact of this stay on parents and their healthy children for whom no continuous aftercare services are offered. This study aimed to capture the post-stay experience and needs of parents after this traumatic event so that they could return to family and everyday life.

**Methods:**

This qualitative descriptive study was conducted in collaboration with four pediatric intensive care units in Switzerland. It included parents whose children had fully recovered after a stay and who did not require continuous medical follow-up. All children were hospitalized in the PICU for at least 48 h. Data were collected through narrative pairs (*n* = 6) and individual interviews (*n* = 8). Interviews were audio recorded, transcribed, coded inductively according to Saldaña, and analyzed.

**Results:**

The results showed three related phases that influence each other to restore normality in daily life: Trust and inclusion in the treatment process during the stay (1), processing after the stay (2), and returning to everyday life (3).

**Conclusion:**

Follow-up meetings should be available to all parents whose children have been hospitalized in the PICU. In particular, it should also be available to parents whose children have fully recovered and no longer have any medical disabilities.

## Introduction

Medical and technological advances in pediatric intensive care have increased the survival rate of seriously ill children [1]. A child is critically ill when life is threatened through the dysfunction of one or more organs, such as the heart, lungs, or brain, due to disease or accident, and treatment in a pediatric intensive care unit (PICU) is necessary [2]. In the USA, approximately 230,000 critically ill children are treated in a PICU annually [3]. According to the minimum data set of the Swiss Society of Intensive Care Medicine (MDSi), 5034 children aged 0 (37 completed weeks of pregnancy) to 16 years were admitted to a PICU in Switzerland in 2020. For parents, their critically ill child staying in a PICU can be a significant and traumatic experience [4]. Often, upon admission to the PICU without any preparation, the rapid change in the family situation must be accepted, and everyday family life must be reorganized [5]. Fear, insecurity, worry, uncertainty, sadness, helplessness, anger, powerlessness, and feelings of guilt are challenging for parents of critically ill children [6]. Parents are suddenly confronted with a loss of control over their parental role [7]. Of parents, 84% exhibit symptoms of acute stress disorder (ASD) after their child is admitted to a PICU [3]. From this, according to Nelson et al. [3], 10–21% of parents develop post-traumatic stress disorder (PTSD). These symptoms are triggered by increased psychological, physical, and emotional stress levels [8]. The measured stress level of parents [9] at admission and during their child’s PICU stay is high, and parental stress is even higher after the PICU stay [10]. Furthermore, critically ill children may develop post-intensive care syndrome (PICS-P) after a long intensive care stay [1]. According to Ekim [1] and Essens et al. [11], critical illness can result in cognitive, psychological, and physical deterioration in children. These symptoms often manifest long after hospital discharge. Both these factors have long-term consequences for the family and represent an increased burden on daily and family life. Currently, international focus on the long-term consequences of PICU stay for children and parents is minimal [5]. However, when children die in a PICU in Switzerland, parents receive an invitation from their respective institution for a follow-up discussion. Such discussions provided them with lasting support during the process of coping and grieving [12, 13]. For parents of children who have been cured or are on the road to recovery, no offer is provided in the form of a follow-up meeting in Switzerland. In particular, parents with children who cannot automatically be assigned to a follow-up care service owing to their illness fall into the care gap. After discharge from the PICU, the parents were positive regarding the possibility of being invited for a follow-up appointment. However, some parents did not want to take advantage of this offer [14]. Among the most traumatized parents, such interventions have been shown to reduce long-term distress [15]. In an adult intensive care setting, a follow-up meeting showed significant benefits for patients and their families in terms of reducing PTSD and ASD [16]. It helps patients and their families to better understand and process the time spent in the new PICUs. Aftercare services also have the potential for further development in other countries in Europe and worldwide. Little emphasis has been placed on debriefing in aftercare settings [17], although debriefing for parents and children is necessary [18]. Memory and long-term consequences manifest themselves in parents even 2 years after such a critical event [19]. In this process, the increased experience of stress affects daily and family life through powerlessness, fear of loss, and coping with lingering trauma [18]. Therefore, offering targeted interventions to families with children who do not receive aftercare services is necessary [20]. Research conducted to date has focused on parents and children participating in ongoing aftercare services following a stay in the PICU for acquired or chronic conditions [18]. Little is known about the traumatic experiences of those parents whose child has been treated in a PICU for a health-related life-threatening illness and has made a full and permanent recovery. This study aimed to investigate how parents experienced the period following their critically ill child’s stay in an intensive care unit and the impact of traumatic events on the family and daily lives of children who have fully recovered and no longer have any disabilities.

## Method

### Design

The experiences of parents after their child’s stay in a PICU who did not participate in any care program are examined using the qualitative, descriptive method [21, 22]. This qualitative descriptive study exploratively oriented to generate data that describe the effects of traumatic experiences from affected parents’ subjective perspective on family and everyday life [23]. This study design is particularly suitable for answering the research question, to stay close to and describe participants’ experiences, so that certain patterns and characteristics of parents’ individual lifeworld can be collected, described, and interpreted.

### Setting/population

Parents whose children were critically ill and hospitalized in one of four out of eight PICUs in Switzerland were recruited for the study. Parents whose child therapy in the PICU was unavoidable due to the illness were included. Children had to be fully recovered, not embedded in continuous follow-up care after their stay and showed no further chronic disease progression. Owing to the emotional impact on the parents and considering the arrival in the family routine, the time since discharge should be at least 2 months. Parents whose children died during their stay were not included in the study. Similarly, the parents must speak and understand German.

The study excluded parents whose children were younger than 37 weeks of gestation and older than 16 years old at the time of their stay. Especially children younger than 37 weeks of gestation and their parents have different needs in a postnatal follow-up [24]. The length of stay in the PICU was at least 2 days for all children.

### Recruitment and sampling

Eligible parents were recruited in collaboration with four PICUs in Switzerland. After the PICUs agreed to help recruit participants, a contact person at the respective institution wrote to potential parents who could be considered for the research project. The parents were contacted through mail with relevant study information and the contact details of the first author. Subsequently, parents had the opportunity to contact the first author directly to register for participation. Due to a lack of feedback, the study information was disseminated via LinkedIn and a snowball system. This enabled further parents to be recruited. During the first contact, the first author provided detailed information regarding the study project. This was done via mail or telephone. After obtaining verbal consent from the parents, interviews were conducted. To achieve heterogeneity and maximize variation in parents’ lifeworld experiences and descriptions, parents with experience in another country should be included [25].

### Data collection

Interviews were conducted between November 2021 and February 2022 using the narrative method. The interview method is particularly well-suited for qualitative health and social research. It focuses on the narratives of the interviews. Thus, the lifeworld, associated experiences, and events can be explored [26]. Interviews were conducted using a narrative approach [24–26]. Prior to the start of the interview, the interviewees signed a consent form stating that they had received the study information and agreed to be interviewed. As a supplement, sociodemographic data, such as the age of parents, family members, length of stay of the child in the PICU, and age of the child, were collected. The interviews were conducted in three phases [26]. In the introductory phase, open-ended questions are asked to determine the direction of the topic. “*When they think of being in the PICU, what is the first thing that comes to mind?”* The main and narrative phases opened up the opportunity for parents to tell their own stories regarding critical events and related experiences after their critically ill child’s stay in a PICU. In doing so, the interviewer noted possible questions during the inquiry phase. The closing phase provided interviewees with the opportunity to explain their concerns, questions, or requests [27, 28]. During the interviews, additional field notes, observations, nonverbal communication, and home environments were documented and included in data collection. The interviewees determined the interview location for each case. Paired or individual interviews were conducted based on the parents’ choices. The interviews were digitally recorded, transcribed verbatim into standard language using MAXQDA (2020), and edited. The interview guide was tested during the first interview. Data saturation was evident from the point at which the previous interview provided the same data. The more interviews, the lower the contribution of the subsequent interview [29]. Personal and health-related data were anonymized so that no inferences could be made regarding the study participants, locations, or events. These data served as background information and supplement for data analysis. Sociodemographic characteristics are presented as totals and means in the results. Data were stored in a secure folder in the Zurich University of Applied Sciences (ZHAW) database.

This study doesn’t fall within the scope of Swiss Human Research Act. A declaration of non-responsibility has been made available (BASEC No. Req-2021-009959). The study participants have signed an informed consent. The research process was based on the Swiss guideline on research involving humans published by Swiss Academy of Medical Sciences (SAMW).

### Data analysis

The transcribed interviews were processed and analyzed using the coding method described by Saldaña [30] based on an exploratory approach using qualitative analysis software (MAXQDA Analytics Pro 2020). For the processing of the data, the researcher applied in vivo [31, 32] and initial coding (open code) [32]. This step included the first cyclic coding [30]. Subsequently, second cyclic coding was applied. The identified codes (words and sentences) were summarized, compared across cases, and classified into patterns and subcategories. Certain categories are formed from these subcategories [23].

The study complied with the quality criteria [33]. The obtained and processed interview data were archived for credibility. The transferability was based on the detailed results. Traceability can be guaranteed at any time because the research process has been continuously documented. The last author constantly reviewed the research process, and individual steps were critically observed and discussed.

## Results

A total of 14 interviews were conducted with parents, 14 mothers and six fathers, six of which were couple interviews. In eight interviews, only the mothers participated in each case. Nine interviews occurred in the home environment, three online, and two at the place of work. The interviews lasted between 46 and 97 min. Further information on the sociodemographic data of the study participants is shown in Table 1. One interview was included as a comparison in the data analysis, although the child was not hospitalized in a PICU in Switzerland (Table 1). The experience of having a child with a life-threatening illness was the focus, not the location of the PICU.

**Table 1:** Residence information and sociodemographics of study participants

	*n* (%)	Md
Number of interviews	14	
Participant interview Mothers Fathers Parent pairs	20 14 (70) 6 (30) 6 (30)	
Age of parents (years)		
Min Max	28 49	43
Parents’ highest education		
Basic vocational training Higher vocational education University degree University degree	4 (20) 6 (30) 7 (35) 3 (15)	
Length of stay (days)		
Min. Max.	2 42	7
Age of the child during PICU stay		
1–7 days (from 37 completed weeks of pregnancy) 8 days – 1 month 1 month – 3 years 3–16 years Min (days) Max (years)	5 3 0 6 1 13	
Diagnosis		
Birth defect Trauma Neurosurgical Infections/sepsis	7 2 1 4	
Exit PICU since (months)		
Min. Max.	7.5 120	18

The analysis revealed three phases that the parents went through during and in returning to everyday life. Patients experience this process differently depending on the disease occurrence. Each phase contained its own phenomena, was connected to the previous phase, and influenced their returning to everyday life. A certain level of normality is required so that everyday life can be lived as usual. In the first phase of their stay, parents required trust and inclusion in the treatment process. Thus, they can find a way to process in the second phase, as they find in the third phase, and return to familiar everyday life. Familiar procedures and rituals that were in place before their stay in the PICU supported the family back into a new normality of everyday family life.

(Fig. 1)

**Fig. 1:**
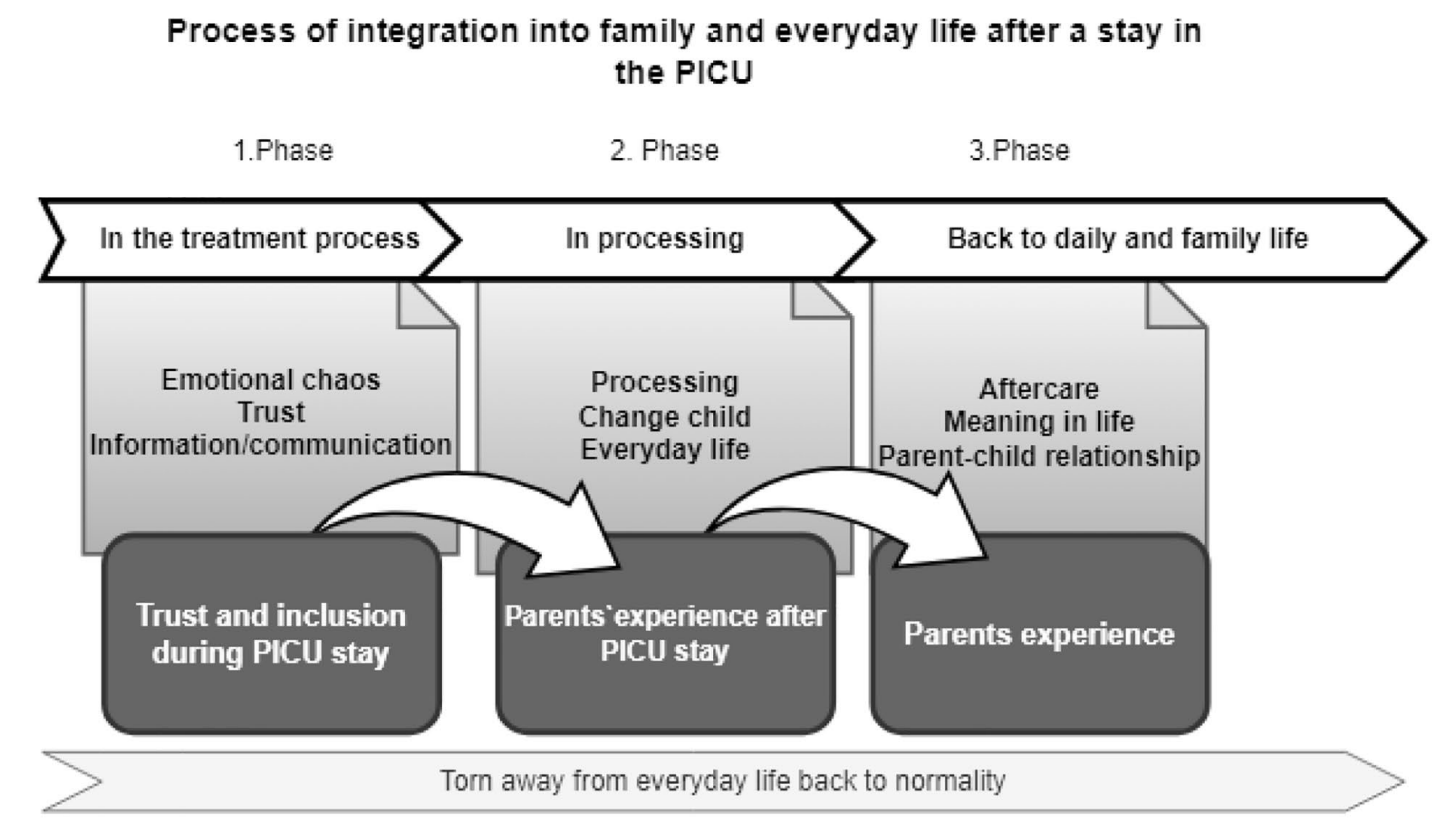
Process of integration into family and everyday life after a stay in the pediatric intensive care unit

### In the treatment process: trust and inclusion during the PICU stay

The shock experience of their child’s admission to the intensive care unit leaves parents in an emotional chaos of fear, helplessness, and uncertainty. The information and communication behavior of the treatment team influenced the parents’ trust as well as their involvement in the treatment process, thus shaping their memories of the stay.

#### Emotional chaos

Memories of the stay were present in many of the parents. The combination of positive and negative feelings between hope and fear is emotional. Parents felt alone from time to time and liked a person to support them. Most were well cared for in the intensive care unit, while some were over-cared for. They described the treatment team as saviors for their children. Often, parents had no choice but to wait until their child was well. They described their stay as the worst they had experienced. Unpreparedness triggers stress, fear, and uncertainty.

*“[…]In principle, if there are different degrees of hardness, for those affected that is irrelevant whether there is even worse! And only because one classifies that medically as harmless, or has a good prognosis, that does not mean automatically for the concerning that they can classify that also exactly the same! " (Father_Interview_11, pos. 123)*.

The parents reported that they felt the ICU was like an island. During their stay, they felt massively at the mercy of the situation, were unable to categorize it, and needed the trust and empathy of the treatment team.

*“ […] therefore, somehow the first moment when I have been in the intensive care unit or also, when I heard, was for me rather a relief.” (Father_interview_1, pos. 5)*.

The fear for their own child was great, and the parents could hardly understand what was happening. Parents of newborn children could not get involved in a big emotional relationship from the beginning because of the fear of losing the child.

#### Trust

The dependence on parents to leave their child in the hands of strangers has led to a loss of control and changed the parental role. Through the involvement of the treatment team, parents were able to better understand and accept the situation. This gave them a positive feeling, and more recovery phases existed. Thinking rationally was often very difficult and parents felt stressed. They were overwhelmed when they had to make decisions regarding their critically ill children. For many parents, everything was very surreal, and the parents were in an emotional state.

*“But I also don’t know, it didn’t exactly repel me, but I realized so I’m……. it had been a bit of the overload. I just let it wash over me and thought, I’ll adjust to it. Emotionally neutral, but so in a weird state of limbo.” (Father_Interview_2, pos. 3)*.

Moving from the PICU to another unit often created a new situation that made parents more anxious and perceived it as a stress factor. The flow of information took longer, and childcare was no longer continuous. Consequently, parents rarely allowed short breaks. They often did not take time off because they were plagued by guilt regarding leaving their children alone.

*“That’s the absolute horror, when you have to leave the little one behind and you can’t even be near quasi.” (Mother_ Interview_7, pos. 6*.

#### Information and communication

Adequate and continuous information was helpful in facilitating a patient’s stay. Unresolved concerns regarding the course of the disease were burdensome, making it difficult to return home afterward. Parents required the same level of information regarding their child’s disease processes, which did not mean that everything was always understood immediately. The timing of the information delivery is crucial. Parents in the ICU usually feel better informed. Being in the middle of an action than waiting at home is easier.

*“For me it was perhaps easier… yes… I sat there, I saw it, it is looked at, although there has been no improvement for a long time, relatively long, because then the doctors have already discussed if it does not really get better, we have to look. I could be there[…]”. (Mother_interview_12, pos. 90)*

Parents need a consistent treatment team. With the change in staff, uncertainty increased. Good conversations and small talk were not to be missed. Parents described the nurses as providing important emotional support. Thus, the parents felt that they were part of the entire process. Their opinions were listened to, and some were perceived as a whole-family system. Most parents receive psychological support from their institutions. This offer often came at the wrong time, or they did not feel the offer was necessary. The assignment of guilt by the treatment team burdened parents. Support from family and friends was very helpful to the parents. However, the continuous need for information from family members and friends who described the situation as stressful was burdensome.

### In the process of adaption: parents’ experience after the PICU stay

In this phase, processing has to occur and simultaneously accept that everyday life had changed from one moment to the next. It should also be perceived that the child has changed. Simultaneously, the parents experience a familiar environment and should thus regain normality in the familiar everyday life or become used to the new everyday life.

#### Everyday life

Some parents struggled because everyday life was no longer the same as it used to be. Physical and emotional exhaustion was initially part of everyday life. A period of a state of emergency that resolved at different speeds existed. One mother provided an impressive account:

*“Yes, yes we were really pulled out and put back in.” (Mother_interview_8, pos. 111)*.

One mother wanted to be at home and do things around the house. This type of stress affects the entire family. Support from the environment helped her considerably. Others enjoyed having no obligations associated with the disease and appreciated being a parent. Involving siblings and allowing them to participate is challenging for some parents. They wanted to do justice for all children and not give preference to those who are sick.

#### Processing

The time after the intensive care admission triggered different emotions in the parents. The difficulty in starting life and being able to take a healthy child home triggered feelings of happiness and gratitude, but also made them reflective and sad.

*“[…] my husband had taken the whole thing differently. He found hey so, what have you, she is now there, and she is now healthy and found I . yes…. And at some point I thought, come on, I think I’d rather stay alone with this feeling and say nothing more, because what’s the point?” (Mother_Interview_3, pos. 8)*.

Parents then often sought support in the form of alternative healing methods, psychological support, or underwent trauma therapy. Others did not seek help because they did not want or need it. Coping strategies were differentiated based on experience. Photo books were created: one with hospital photos and the other without. Diaries were written to support this coping process.

*“ Processing may happen even if it is still current.” (Mother_interview_8, pos. 108)*.

After their stay, the parents needed someone to confirm that everything was fine. They had to take action on their own because it was only afterward that many questions arose that could no longer be answered.

*“When I then began to realize what had happened, I suddenly had uh many questions. And then it is a bit difficult to ask them. “(Mother_interview_5, pos. 6)*.

Moving from the PICU to another unit often created a new situation that made parents more anxious and perceived it as a stress factor. The flow of information took longer, and childcare was no longer continuous. Triggers, such as pictures or music, often cause strong emotions, such as sadness and listlessness, without warning. Parents who had experienced a life crisis were able to apply the coping strategies that they had learned. They experienced the coping process more consciously and sought support. Parents exhibited increased anxiety after the residence, suggesting that something bad might happen to the child again. They worried regarding the effects of medications received in the PICU on the child. They made doctor appointments more quickly when the child had headaches, knee pain, or colds, fearing that it could be something serious again. This also caused emotional stress. Some parents were aware of this; however, they still made appointments for reassurance because they did not want to miss anything. When a phone range or an ambulance drove, uncomfortable feelings arose again. Even a year later, some parents experienced physical symptoms, such as headaches, dizziness, or body tremors. These could not be suppressed. Mothers and fathers often describe a state of exhaustion after their stay when the child was well again. Some parents talked a lot regarding the subject because it helped them in processing, while others simply wanted to stop discussing the subject. Still, others did not have the courage to seek help because they did not have the strength. Taking time off for a vacation was seen as positive by some who considered it a step into a new daily routine. Offers of self-help groups were accepted by some parents for support.

#### Change in the child

Children reacted differently depending on their age. Newborns were alert and awake and required physical proximity. At this age, the parents experienced their children as being strong willed. Infants who had sleep problems or were afraid of darkness were quick tempered or initially quiet. The children wanted to return to their routines quickly. Adolescents did not want a special status at school. The children coped well with the physical restrictions at the beginning and accepted them, although it often annoyed them to be not allowed to do everything. Panic attacks or headaches were also signs that parents were not previously aware of in their children. Likewise, the question was often whether it was normal for the child to react this way during puberty or it had something to do with the disease.

### Back in everyday and family life: the experience of parents

Parents wanted to return to their usual daily and family lives. In the process, it became apparent that they were questioning the meaning of life and the changed parent–child relationship. To find their way back to the new normality, parents could have benefited from an aftercare program at this point.

#### Meaning in life

The parents described how other values became important in their lives. They spend more time with their families and are more active during their free time. They also had a different view of the world and did not understand people’s discussions regarding trivialities or their emphasis on them. The new PICU stay also revealed new perspectives that would not have had the child undergo this course of illness. Fathers reported that they had become more emotional since their stay, viewed the experience as an opportunity, and embarked on new projects. Parents learned from what they had experienced but at different rates. The event strengthened them as families, brought them closer together, and normalized their lives.

*“Yes maybe, it had been the normality too, the normal routines, maybe the school and everything. The normal.“(Mother_Interview_8, pos. 118)*.

These relationships have been tested extensively. Many parents reported that they managed their time together well. The major challenge was that everyone felt the crisis was different.

*“If someone tells you that the accident has already welded you together- you can’t say that- we have borne this together yes, but it is sometimes not so easy” (Father_Interview_14, pos. 115)*.

#### Parent–child relationship

Parents have since developed closer relationships with their children. The relationship between fathers and sons developed positively. The newborn parents reported special relationships in the form of physical and emotional closeness. Parents play an important role because they can influence their children positively or negatively.

#### Aftercare

Although the children were doing well again today, many questions did not arise until weeks or months after their stay. Parents reported that they usually did not know where to voice their concerns or ask questions that were often bothersome. They felt the need to discuss their experiences again with the specialists. Particularly regarding the child’s behavior, many questions arose that unsettled them. Clarifying the discussions with experts would have been helpful. Furthermore, family planning was an issue for some parents. As certain issues are not clarified after birth, this decision is not always easy. One pair of parents did not want any more children after birth even though they had not yet completed their family planning.

## Discussion

This study focuses on parents’ experiences after their child has been hospitalized as critically ill in the PICU and may subsequently return to being without continuous medical care. This novel study demonstrated that regardless of the child’s admission diagnosis, severity of the disease and subsequent course of health and illness, comparable phenomena occur in everyday and family life [18].

The phases that parents underwent differently during and back into family and daily life were interrelated and influenced the physical and emotional well-being of the entire family.

During their stay, building trust and being involved in the treatment process through specific information and adapted communication were important for the parents [34]. Fear of loss, worry, uncertainty, and helplessness were also prominent among parents whose children were hospitalized in the new PICU for only 2 days [17, 19, 20]. Thus, a diagnosis cannot predict how parents cope with traumatic experiences. Individual needs and experiences of this event shaped parents differently. The treatment team, particularly the nurses, played an important role in accompanying critically ill children and their families regardless of the diagnosis or course of the disease. Most parents had to build up trust and a relationship anew when changing departments and, in doing so, had difficulty adjusting to the changed situation.

Processing after the stay was also experienced differently and characterized by fear, uncertainty, sadness, and physical weakness. Life in the social environment became more difficult when it was not understood why parents felt the way they did. The environment expected carefree and happy parents when their children had fully recovered and had no consequential damage. As a result, parents no longer dared to speak regarding this traumatic event and were thus emotionally weakened. Many parents realized this and when they required help. They sought psychological support or helped themselves with other healing methods such as trauma therapy or homeopathy.

In everyday life, often weeks or months later, parents begin to realize what has happened. At that time, parents ask many questions that they could not answer. At that time, they lack the contact they could have turned to because they were not involved in the aftercare program. An offer of care is warranted for these parents and children, just as it is for children with chronic courses and long stays [18]. This intensity depends on the needs of the parents. A follow-up can clarify many unanswered questions and uncertainties [35] and prevent additional doctor visits. The issue of PICS-P is also evident in recovered children. These children also show short- or long-term changes in behavior or are accompanied by physical symptoms such as fatigue, headaches, and insomnia [18]. Parents usually accept invitations for a debriefing. Having a competent contact person to whom they can turn with questions in a low-threshold manner is important. A short phone call often answers many questions.

### Strength and limitations of the study

The strength of this study lies in the recruitment of different PICUs in Switzerland. Comparable and meaningful results have shown that not only the institution but also the experience plays a central role. A limitation of the present study is the difficulty in recruiting the study participants. The reasons for this were the limited possibilities of the first author to make parents aware of this research project during their stay. Parents probably felt the need to not have this experience replayed assuming that the child would accept it. Also, only parents who lived in the German-speaking part of Switzerland were surveyed. Culture could also play a role in a further study, as every culture deals with such emotional stress differently. However, this was not the aim of the research question, but would be an important aspect to take into account for further research. The timing of the interviews after the stay was very different but did not affect the quality of the results. Likewise, no explicit data was collected in the form of the severity of the disease during the stay in the PICU, as the parents were recruited after the stay.

### Recommendation for practice and research

This study clearly demonstrated the need for a low-threshold aftercare service following a stay in the intensive care unit. None of the study participants were involved in continuous medical aftercare. The severity of the illness at the time of hospitalization was not decisive, but what a PICU stay triggered in the parents with a healthy child. This shows that a gap exists in care and that aftercare services could contribute to increasing the psychological and physical well-being of parents. In this context, measuring the stress levels of parents and their children is an important factor in offering suitable aftercare services. Furthermore, institutions and healthcare personnel should be made more aware of parents’ needs after their stay in PICUs. The recommendation here is to offer a follow-up meeting for parents as well, where they can decide individually whether they would like to accept an invitation or not, for example.

## Conclusion

Parents underwent the same process regardless of their child’s diagnosis or length of stay in the new PICU. Parents whose child was hospitalized in an intensive care unit in another country (South America) went through the same stages. The fear and uncertainty that their child might die was also at the forefront. Parents’ trust and involvement in the treatment process have an impact throughout processing and normal everyday life. The study clearly showed that even parents whose children were in the new PICU for only 2 days suffer equally from long-term psychological distress. Most of the parents and their families recovered well and returned to their normal daily lives. They found ways of processing this experience through self-efficacy. However, this experience will continue to accompany them as a family, each in their own individual way.

## Data Availability

The datasets generated and analyzed during the current study are not publicly available due to data protection in accordance with Swiss standards. Although participants have consented to the data being processed, the consent does not cover the further use of the data. Moreover, our raw data are in the form of transcribed and anonymized interviews and only available in German.
